# Evolution of acquired resistance in a ROS1^+^ KRAS G12C^+^ NSCLC through the MAPK pathway

**DOI:** 10.1038/s41698-023-00349-0

**Published:** 2023-01-23

**Authors:** Katherine Priest, Anh Le, Amanuail Gebregzabheir, Hala Nijmeh, Gregory B. Reis, Melanie Mandell, Kurtis D. Davies, Carolyn Lawrence, Emily O’Donnell, Robert C. Doebele, Liming Bao, Dara L. Aisner, Erin L. Schenk

**Affiliations:** 1 Division of Medical Oncology, University of Colorado - Anschutz Medical Campus, Aurora, CO USA; 2 Department of Pathology, University of Colorado - Anschutz Medical Campus, Aurora, CO USA

**Keywords:** Non-small-cell lung cancer, Cancer therapeutic resistance

## Abstract

Patients with metastatic NSCLC bearing a *ROS1* gene fusion usually experience prolonged disease control with ROS1-targeting tyrosine kinase inhibitors (TKI), but significant clinical heterogeneity exists in part due to the presence of co-occurring genomic alterations. Here, we report on a patient with metastatic NSCLC with a concurrent *ROS1* fusion and *KRAS* p.G12C mutation at diagnosis who experienced a short duration of disease control on entrectinib, a ROS1 TKI. At progression, the patient continued entrectinib and started sotorasib, a small molecule inhibitor of KRAS p.G12C. A patient-derived cell line generated at progression on entrectinib demonstrated improved TKI responsiveness when treated with entrectinib and sotorasib. Cell-line growth dependence on both ROS1 and KRAS p.G12C was further reflected in the distinct downstream signaling pathways activated by each driver. Clinical benefit was not observed with combined therapy of entrectinib and sotorasib possibly related to an evolving *KRAS* p.G12C amplification identified on repeated molecular testing. This case supports the need for broad molecular profiling in patients with metastatic NSCLC for potential therapeutic and prognostic information.

## Introduction

Lung cancer remains the leading cause of cancer-related deaths for men and women in the United States^1^. For patients newly diagnosed with metastatic non-small cell lung cancer (NSCLC), treatment decisions are based on broad molecular profiling of the tumor including tissue sequencing and oncogenic fusion and amplification detection^2^. In a subset of these patients, molecular testing will identify targetable alterations such as an epidermal growth factor receptor (*EGFR*) mutation or ROS proto-oncogene 1 (*ROS1*) gene fusion. Patients with NSCLC bearing a targetable alteration, such as a *ROS1* fusion, can experience durable, prolonged disease control with molecularly targeted, tyrosine kinase inhibitors.

For the 1–2% of patients diagnosed with NSCLC positive for a *ROS1* fusion (ROS1^+^), a current first-line therapy option, entrectinib, results in a disease response in the majority of patients with expected survival measured in years^3^. Despite effective TKI therapy, nearly all ROS1^+^ NSCLC develop treatment resistance and disease progression occurs. At time of disease progression, repeat tissue or cell free DNA molecular testing are frequently pursued as some mechanisms of TKI resistance can be inhibited with other clinically available therapies^4^. Acquired mechanisms of resistance to ROS1 TKIs include ROS1-dependent mechanisms, such as *ROS1* kinase domain mutations, or ROS1-independent mechanisms including bypass signaling through the mitogen-activated protein kinase (MAPK) pathway. Acquired Kristen rat sarcoma virus (*KRAS*) mutations or amplifications have been reported in a subset of patients with ROS1^+^ NSCLC^5^. Resistance arising through *KRAS* mutations, specifically *KRAS* p.G12C mutations, are of particular interest given the development and clinical availability of KRAS G12C inhibitors^6^. These concurrent alterations in other targetable drivers are primarily noted at the time of TKI resistance, and rarely at diagnosis^7^. Here we report on a patient with metastatic lung adenocarcinoma bearing both a *ROS1* fusion and *KRAS* p.G12C mutation at diagnosis. Despite molecularly targeted therapies for both oncogenic alterations, the patient did not experience clinical benefit from therapy likely due to evolving outgrowth of a *KRAS* p.G12C bearing clone and eventual *KRAS* p.G12C amplification. This case underscores the importance of broad molecular profiling in advanced NSCLC both at the time of diagnosis and at treatment milestones such as progression to personalize therapy and outcome expectations for patients.

## Results

### Clinical history

**Fig. 1:**
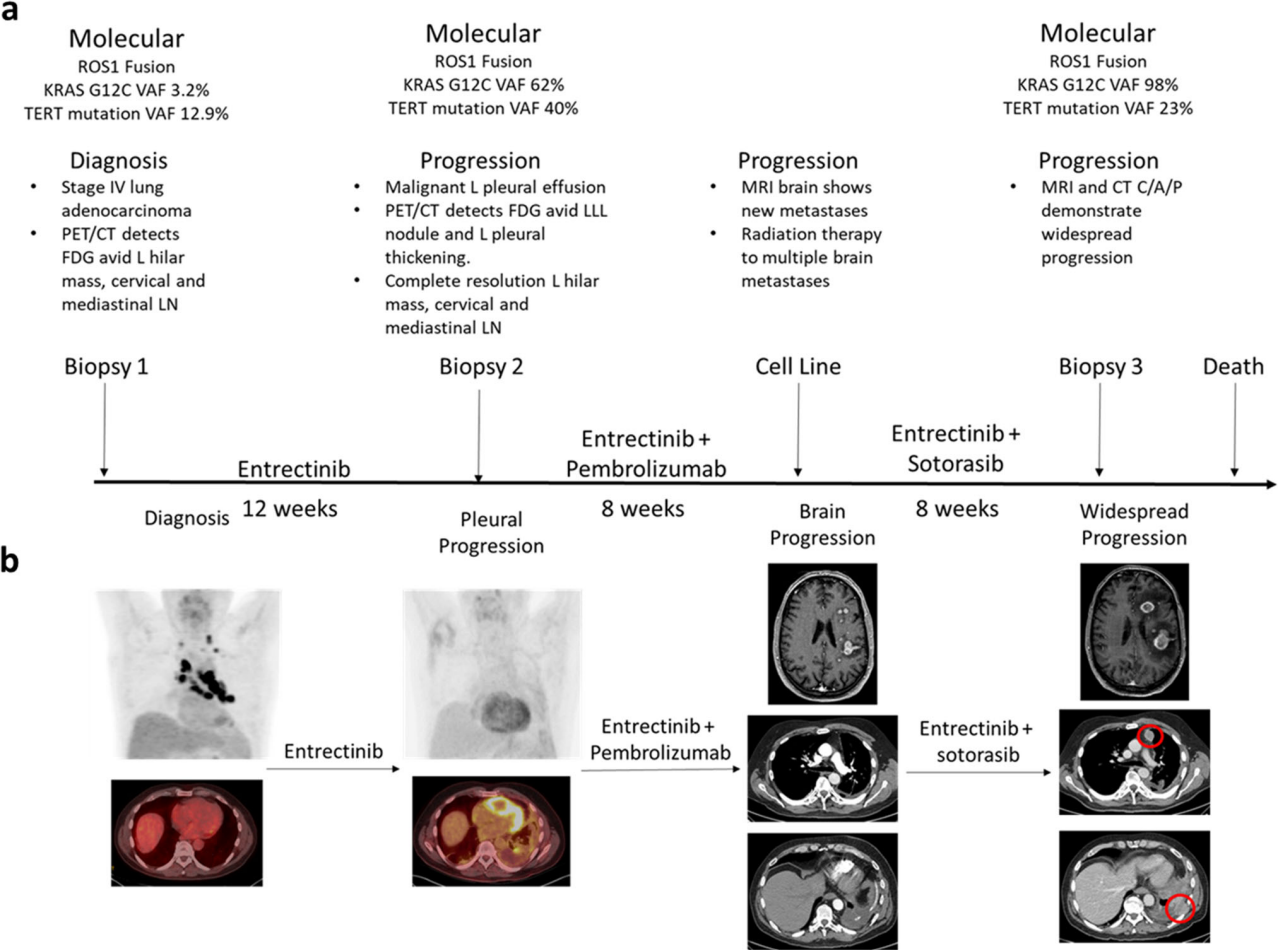
Patient clinical course. **a** Timeline of the patient’s diagnosis, treatment, and molecular testing results. **b** Radiographic changes associated with patient’s clinical course.

A 57-year-old man with a smoking history of less than 5 pack-years presented with bilateral leg pain due to deep venous thrombosis in both legs. A computed tomography (CT) scan of the chest demonstrated multiple, bilateral acute and subacute pulmonary emboli and a left hilar nodal mass with left-sided mediastinal lymphadenopathy. A bronchoscopy with endobronchial ultrasound guided biopsies demonstrated adenocarcinoma consistent with a lung primary at station 11L (Fig. 1a). While holding his anticoagulation after the procedure, the patient presented with expressive aphasia and was found to have an acute middle cerebral artery infarct. During his recovery, molecular testing on the biopsy reported a *ROS1* fusion identified by fluorescence in situ hybridization (FISH), and next-generation sequencing (NGS) of tumor DNA identified a *KRAS* p.G12C mutation and a telomerase reverse transcriptase (TERT) promoter mutation (c.-124C>T). At diagnosis variant allele frequency (VAF) determined was 3.2% for *KRAS* p.G12C and 12.9% *TERT* mutation. Positron emission tomography (PET) demonstrated FDG avid left hilar mass and extensive adenopathy throughout the neck and mediastinum (Fig. 1b). Magnetic resonance imaging (MRI) of the brain showed no evidence of intracranial metastases. The patient was started on first line entrectinib.

Approximately 3 months later, the patient developed a left sided pleural effusion and a thoracentesis was positive for metastatic lung adenocarcinoma. Molecular testing again demonstrated a *ROS1* fusion by FISH and a *KRAS* p.G12C mutation. Allele frequency of *KRAS* p.G12C was 62% compared to 40% for the TERT mutation suggesting outgrowth of clones bearing a *KRAS* p.G12C mutation. Subsequent tissue sequencing by NGS at our institution did not detect a *ROS1* kinase domain mutation and the fusion event was determined to be TPM3(ex8)-ROS1(ex35). PET imaging revealed near complete resolution of the previously FDG avid left hilar mass and neck and mediastinal adenopathy but a new FDG avid posterior left lower lobe nodule and left pleural thickening was noted (Fig. 1b). At an outside institution, the patient was started on pembrolizumab along with continued entrectinib.

One month later, the patient presented to our institution with progressive, expressive aphasia. MRI of the brain demonstrated new multifocal supratentorial lesions predominantly within the left frontal and left parietal lobes (Fig. 1b). The patient received radiation therapy to the brain lesions. Approximately 4 weeks later, the patient started sotorasib plus entrectinib and pembrolizumab was discontinued. One day after initiation of dual targeted therapies, the patient required hospitalization for progressive hypoxia due to bilateral pulmonary emboli and new community acquired pneumonia which were not thought due to his previous immunotherapy or his dual targeted therapies. During his treatment course on both targeted therapies, he was frequently evaluated by his primary oncologist and clinical team. The patient did not experience adverse events that could be considered related to combination therapy. Specifically the patient did not require antiemetic therapy and had stable weights during his evaluations. No liver function test or electrolyte abnormalities were noted and creatinine was normal. Approximately 2 months later, the patient returned with progressive worsening of his baseline aphasia along with 1 week of right arm weakness and paresthesias. MRI imaging of the brain showed interval progression in 3 hemorrhagic lesions within the left cerebral hemisphere (Fig. 1b). CT imaging of the chest, abdomen and pelvis showed progression on the left pleural disease with new abdominal lymphadenopathy. During the hospitalization, a biopsy of a left pleural mass was performed and molecular testing demonstrated a *KRAS* p.G12C VAF of 98% compared to 23% for the *TERT* mutation. While hospitalized, the patient was experiencing a clinical recovery of his speech and right arm function. Anticoagulation was restarted with argatroban due to a 40% decrease in platelet counts over 5 days with recent low molecular weight heparin exposure. The day of argatroban initiation, the patient experienced acute clinical deterioration while ambulating was noted to have fixed and dilated pupils, and died likely due to an acute intracranial hemorrhage.

### Cell line characterization

**Fig. 2:**
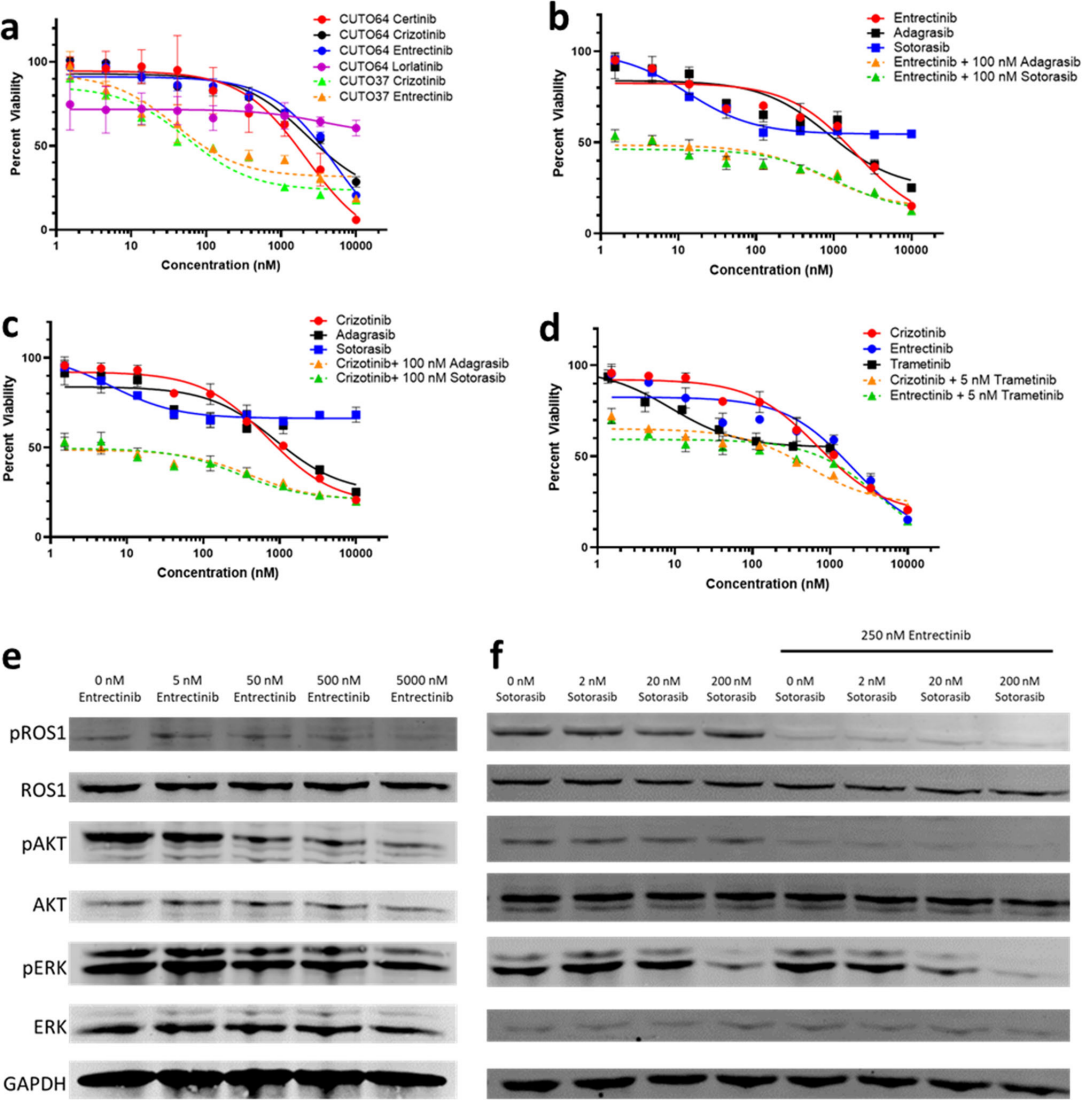
CUTO64 is resistant to single agent ROS1 inhibition but sensitive to combined ROS1 and KRAS G12C inhibition. **a** CUTO64 is resistant to clinically available 1st line ROS1 TKIs (ceritinib, crizotinib, entrectinib) and 2nd line lorlatinib, in comparison to CUTO37 a patient derived cell line responsive to ROS1 TKIs. Inhibition of KRAS G12C with clinically available sotorasib or adagrasib increases CUTO64 responsiveness to ROS1 inhibition with **b** entrectinib or **c** crizotinib. **d** Inhibition of ROS1 and the MAPK pathway with trametinib increases CUTO64 responsiveness to TKI. Showing the mean ± SD, *n* = 3 biological replicates. **e** Treatment of CUTO64 with entrectinib resulted in pROS1 inhibition and a reduction in downstream Akt activity but had limited impact on ERK signaling. **f** Single agent sotorasib reduced ERK signaling but had no impact on pROS1 or pAkt. The combination of entrectinib plus sotorasib resulted in inhibition of both the MAPK and Akt pathway.

Prior to initiation of sotorasib, pleural fluid was collected for cell line generation (Fig. 1a). The cell line that grew out was designated CUTO64 (TPM3-ROS1) and demonstrated resistance to first line ROS1 targeted therapies entrectinib and crizotinib, with half-maximal inhibitory concentrations (IC_50_) > 2000 nM for both agents in contrast to a ROS1 TKI responsive cell line derived from a different patient, CUTO37 (CD74-ROS1) that has an IC_50_ of 56 nM with crizotinib and an IC_50_ of 39 nM with entrectinib (Fig. 2a). CUTO64 was similarly resistant to ceritinib in addition to second-line lorlatinib with an IC_50_ > 2000 nM for both. (Fig. 2a). CUTO28 (TPM3-ROS1), a cell line derived from a different patient, exhibits sensitivity to entrectinib with an IC_50_ of 8.3 nM (Supplementary Fig. 2) suggesting the ROS1 TKI resistance exhibited by CUTO64 is not due to the ROS1 fusion partner.

As the patient’s clinical testing also demonstrated a *KRAS* p.G12C mutation, we used the combination of entrectinib plus KRAS G12C inhibitors sotorasib or adagrasib which reduced the cell viability compared to either agent alone (Fig. 2b). Entrectinib plus sotorasib resulted in an IC_50_ of 5.8 nM while entrectinib plus adagrasib resulted in an IC_50_ of 7.8 nM compared to single agent IC_50_ of >1900 nM, >2600 nM, and >1000 nM for entrectinib, sotorasib, and adagrasib, respectively. Treatment of CUTO64 with entrectinib and a KRAS G12C inhibitor was synergistic with combination indices (CI) of 0.12 and 0.17 for the combination with sotorasib or adagrasib, respectively. A similar pattern was observed with crizotinib plus sotorasib or adagrasib (Fig. 2c) with an IC_50_ > 1000 nM for each drug individually. Combination of crizotinib and a KRAS G12C inhibitor was synergistic with a CI of 0.16 for crizotinib plus sotorasib and 0.21 for crizotinib plus adagrasib. Inhibition of the MAPK signaling pathway with trametinib, a mitogen-activated protein kinase 1 and 2 (MEK1/2) inhibitor, reduced CUTO64 viability when combined with entrectinib or crizotinib (Fig. 2d).

As concurrent inhibition of ROS1 and KRAS G12C increased cell sensitivity to targeted therapy, we evaluated the signaling pathways downstream of each driver. Treatment of CUTO64 with entrectinib resulted in pROS1 inhibition suggesting resistance was not due to a kinase domain mutation commonly observed in approximately 40% of patients at time of progression on single agent TKI therapy^5^. ROS1 inhibition lead to a dose dependent reduction in downstream protein kinase B (Akt) activity but had limited impact on extracellular-signal-regulated kinase (ERK) signaling at higher drug concentrations (Fig. 2e). Single agent sotorasib reduced ERK signaling but had no impact on pROS1 or pAkt (Fig. 2f). The combination of entrectinib plus sotorasib resulted in potent inhibition of both the MAPK and Akt pathways (Fig. 2f).

### Identification of KRAS amplification through NGS and FISH

**Fig. 3:**
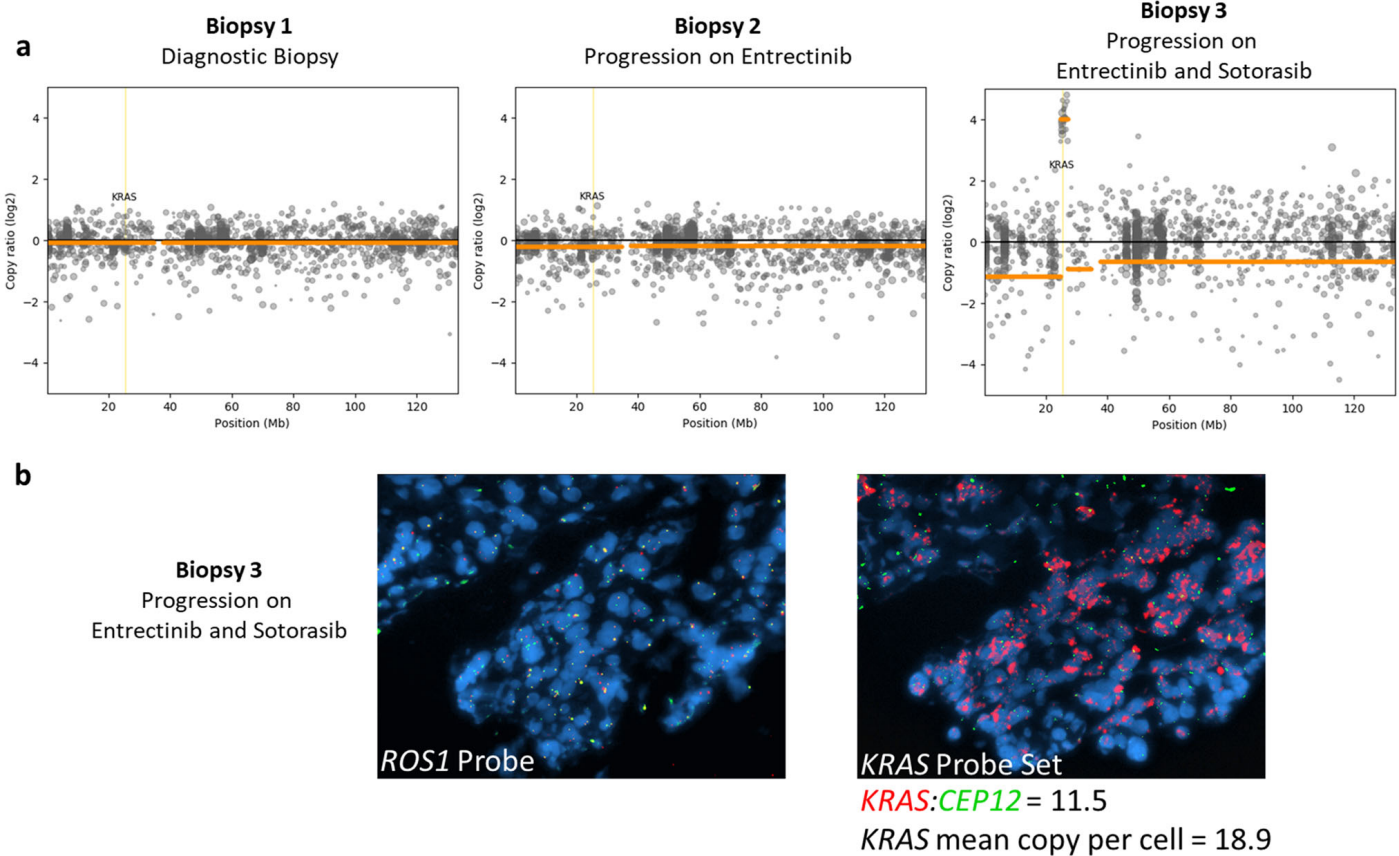
Identification of KRAS amplification at time of patient progression on entrectinib and sotorasib. **a** KRAS copy number alterations identified on sequencing of clinical biopsies at time of diagnosis (left), progression on entrectinib (middle), and progression on entrectinib and sotorasib (right). **b** *ROS1* and *KRAS* FISH on patient biopsy after progression on entrectinib and sotorasib.

In previous studies, *KRAS* amplification has been reported as an acquired mechanism of resistance in patients with progression on ROS1 targeted therapies, in *KRAS* p.G12C positive preclinical models testing sotorasib and in patients receiving other KRAS G12C inhibitors^5,8–10^. Clinical testing on biopsy specimens from the patient demonstrated an increase in *KRAS* p.G12C variant allele frequency in comparison to a passenger mutation in *TERT* over time, suggesting evolving selection of *KRAS* p.G12C co-mutation and amplification as an acquired mechanism of resistance through the patient’s course of therapy (Fig. 1a). Expanded testing with the research-based 498-gene NGS-based approach did not detect *KRAS* p.G12C amplification at diagnosis through our bioinformatics pipeline that includes assessment for copy number alterations (Fig. 3a). After therapy initiation, *KRAS* p.G12C amplification was not detected at time of progression on entrectinib and was readily detectable at time of disease progression on entrectinib and sotorasib. We further verified this amplification with the tissue sample at time of progression on entrectinib and sotorasib by FISH which demonstrated a *KRAS*/*CEP12* ratio of 11.5 and uniform *ROS1* positivity across tumor cells (Fig. 3b).

## Discussion

This case report highlights the critical importance of broad molecular profiling in patients with metastatic lung cancer as co-occurring alterations may inform prognostic discussions and additional therapeutic options^11^. To the best of our knowledge, this case is also the first report of a lung adenocarcinoma bearing both a *ROS1* fusion and *KRAS* p.G12C mutation at diagnosis and the concurrent use of targeted therapies for both drivers. *KRAS*-mediated resistance to ROS1 inhibitors was predicted in preclinical models using crizotinib where a *KRAS* p.G12C mutation was identified in the HCC78 (SLC34A2-ROS1) ROS1^+^ cell line^12^. This clinical case is also consistent with previous work by our group showing primary resistance to TKI therapy in a patient with concurrent *ALK* gene fusion and *KRAS* p.G12C mutation^13,14^. Bypass signaling through the MAPK pathway is a known driver of resistance to ROS1 TKI therapy and ongoing clinical trials are exploring the utility of combining ROS1 TKIs with MEK inhibition^12,15–17^. However, as illustrated by this case, precision therapies targeting driver alterations can be rapidly surmounted by adaptive resistance. Clinical management of systemic progression on a ROS1 fusion targeting TKI can be informed by subsequent molecular testing at time of progression^18^. For this patient, management options included a combination strategy for the known oncogenic drivers or chemotherapy with or without the continuation of targeted therapy. While KRAS G12C inhibition represents a new therapeutic approach for patients with lung cancer, prospective data report response rates of 40.7% in the subsequent line setting and durability of response of approximately 1 year^19^. Retrospective series on pemetrexed-based chemotherapy in patients with *ROS1* fusion positive NSCLC reports a range in disease response from 23.8% to 54.5%^20,21^. Historically, continuing TKI therapy while receiving subsequent line chemotherapy did not improve outcomes but this remains an active area of investigation especially in the era of CNS-penetrant TKIs^22–24^.

Notably even in the presence of a *KRAS* p.G12C mutation, initial therapy with entrectinib resulted in clinical improvement in the patient’s mediastinal tumor burden that persisted until the patient’s death. Studies with CUTO64, derived at time of entrectinib progression, suggests the tumor remained partially dependent on ROS1 as maximal reduction in cell viability was achieved when ROS1 inhibition was paired with MAPK pathway inhibition. Similarly, single agent KRAS G12C inhibitors had limited impact on CUTO64 viability in the absence of ROS1 inhibition. CUTO64 dependence on both ROS1 and KRAS G12C was further reflected in the distinct downstream signaling pathways activated by each driver. However, clinical benefit was not observed with combined therapy of entrectinib and sotorasib likely related to the *KRAS* amplification observed on molecular testing. Clinically, the patient did not have access to KRAS G12C inhibitors until later in the disease course when subsequent line sotorasib was approved by the Food and Drug Administration (FDA). Based on the partial dependence of CUTO64 on both drivers, clinical benefit may have been derived if combined entrectinib and sotorasib were started at diagnosis. Due to the initial response to therapy and clinical considerations, tumor biopsies were not obtained from the same site and the spatial heterogeneity of the obtained samples is a limitation of our study^25^. Molecular testing of samples at times progression may reflect tissue related factors shaping the genomic landscape of the cancer cells in addition to cancer-cell intrinsic mechanisms of therapeutic resistance^25^.

The presence of co-occurring, actionable drivers in NSCLC is uncommon, but the application of NGS testing to clinical care has improved the recognition of this event. In a retrospective series of 3,077 patients diagnosed with NSCLC, NGS testing identified co-occurring, potentially targetable oncogenic drivers in 46 patients, including 16 patients identified prior to therapy initiation^26^. Patients with NSCLC bearing multiple targetable alterations have been reported to experience sustained disease control either through alternating oncogene targeting therapies or combination approaches^26–28^. These reported cases frequently involve *EGFR* mutations alongside *ALK* fusions or *MET* amplifications. Fewer studies have described *ROS1* positive NSCLC with concomitant driver oncogenes, and there has been some discordance in the reported frequency of co-occurring drivers likely due to the method of *ROS1* fusion detection^7,29,30^. NGS testing on 166 *ROS1*^+^ NSCLC without associated clinical information identified 1 patient with *ROS1*^+^ NSCLC with a concurrent *EGFR* L858R mutation and 3 patients with *ROS1*^+^ NSCLC and a *KRAS* activating mutation (Q61R, G12R, G12C)^7^. In the setting of progression on a ROS1 TKI, bypass activation of the MAPK signaling pathway, including *KRAS* activating mutations and amplifications, have also been reported as acquired changes^5^. Pre-clinical studies associated entrectinib resistance with the acquisition of a *KRAS* p.G12C mutation and amplification of both *KRAS* and *FGFR3* in HCC78, an NSCLC cell line bearing a *ROS1* fusion^8^. In this model, entrectinib resistance was associated sustained pERK activation and resensitization to ROS1 inhibition was achieved with the addition of the MEK1/2 inhibitor selumetinib. KRAS amplification is also a reported mechanism of resistance to sotorasib in a patient with *KRAS* p.G12C mutant NSCLC and a patient with *KRAS* p.G12C mutant colon cancer^10^. One potential approach to overcoming *KRAS* amplification is to target upstream or downstream components of the KRAS signaling pathway as is being explored in early clinical trials with SHP2 inhibitors and ERK1/2 inhibitors^31^.

Our data along with other reports highlights the importance of evaluating for alterations in the MAPK pathway as part of a broad assessment at time of ROS1 TKI resistance. In addition to identifying a likely mechanism of resistance, the therapeutic landscape for targeting MAPK pathway alterations continues to expand including clinically available KRAS G12C inhibitors, and ongoing clinical trials testing inhibition of non-G12C *KRAS* mutations as well as downstream targets such as MEK and ERK^32,33^. In conclusion, this case report underscores the need for comprehensive molecular testing of patients with metastatic lung cancer at the time of diagnosis as well as at progression on targeted therapy, as multiple, targetable oncogene drivers may influence treatment choice and expectations for disease control.

## Methods

### Clinical testing

Initial clinical testing was performed through the NeoTYPE™ Analysis Lung Profile (Neogenomics) which analyzes 49 biomarkers through a combination of next-generation sequencing (NGS), fluorescence in situ hybridization, and immunohistochemistry^34^.

Mutational analysis of clinical samples was also performed using a research-based 498-gene NGS-based approach at the University of Colorado (Colorado Molecular Correlates Laboratory). Briefly, total nucleic acid was extracted and mechanically sheared. Sheared nucleic acid was then submitted to library preparation using the KAPA Hyper Prep kit (Roche, Basel, Switzerland). Target enrichment of the library was performed using xGen Lockdown Hybridization probes and kit (IDT, Coralville, IA). Enriched library was then sequenced on an Illumina NextSeq instrument (Illumina, San Diego CA). Raw sequence data was analyzed for mutations and insertions/deletions with a custom bioinformatic pipeline that utilized Novosort for read alignment, GATK/Mutect2 and VarScan2 for variant calling, and Ensenbl Variant Effect Predictor for variant annotation. Copy number alterations were determined using CNVkit^35^.

Dual color FISH assays were performed on 4 µm sections of formalin fixed paraffin embedded (FFPE) tumor specimen with ROS1 break-apart (Vysis, Abbott molecular, and Agilent) and KRAS/12 centromere enumeration (Empire Genomics) probes, using proteinase K enzymatic digestion, or on patient-derived cultured cells. Fluorescent probe signals were evaluated in minimum 50 tumor nuclei per specimen. Specimens were counted as positive for ROS1 rearrangement if 15% or more of cells displayed a split 5’/3’ and/or single 3’ signal pattern. Separation between 5’ and 3’ ROS1 signals of 1 or more signal diameter was required to be considered as a split. KRAS amplification was defined as ratio of KRAS probe to 12 centromere enumeration probe of >2.0.

### Cell line derivation and cell culture and reagents

CUTO64 and CUTO37 cell lines were derived as previously described with patient consent under COMIRB-approved protocol 11–1621 with written consent^36^. In brief, the CUTO64 line was derived from a sample of the patient’s pleural effusion which was applied onto the Ficoll density gradient medium (Histopaque-1077, Sigma-Aldrich # 10771-100 ML) and underwent centrifugation as recommended by the manufacturer. The resulting cell pellet was resuspended in RPMI 1640 media (Gibco # 11875093) supplemented with 10% fetal bovine serum (Atlas Biologicals # F-0500-A) and plated out onto a 25 cm flask. Based on visual inspection of cell morphology, when adherent tumor cells became the predominately established cell type in the culture flask, the culture was subjected to differential trypsinization and mechanical dissociation in order to dislodge the remaining minor population of stromal cells. Cell lines were cultured in RPMI 1640 with 10% FBS and incubated at 37 °C in 5% CO_2_.

### MTS

CUTO64 and CUTO37 cells were plated at a density of 1500 to 2000 cells per well in a 96-well plate and treated with designated drug 24 h post plating. After 72 h exposed to drug, media was changed to remove cell debris and MTS reagent was added to the manufacturer’s recommendations (Promega, G7570). Absorbance was measured on a microplate reader (BioTek) at 490 nm. Non-linear regression curves and IC_50_ were calculated using GraphPad Prism v9.3.1 (GraphPad Software) and CompuSyn v1.0 (www.combosyn.com). The combination index was calculated using the Chou-Talay method with CompuSyn v1.0^37^. Adagrasib, sotorasib, trametinib, ceritinib, crizotinib, entrectinib, and lorlatinib were all purchased from ChemieTek.

### Western blot

Cells were plated in a 6 well plate, cultured to 80–100% confluency and were then treated with the indicated drug(s) and dose(s) for 3 h and processed in parallel. Cells were then lysed with 150 μL of T-PER Tissue protein Extracting Reagent (ThermoFisher) with 1× Halt Protease Inhibitor (ThermoFisher) for 20 min at 4 °C. The lysing solution was collected and centrifuged. The supernatant was collected and stored at −80 °C. The lysate was then diluted 3:1 with 4× Protein Loading Buffer (LI-COR) with 0.1 mM DTT (ThermoFisher). The samples were loaded onto a 10% APS Gel with Chameleon Duo Ladder (LI-COR) and Page Ruler Prestained Plus Protein Ladder (ThermoFisher) and run. Gels were transferred onto a nitrocellulose membrane (LI-COR) and transferred using the Thermofisher PierceG^2^ Fast Blotter as per the manufacturer’s recommendations. Membranes were blocked in 1:1 1× TBS (Research Products International) and Intercept Blocking Buffer (LI-COR) and then tagged with indicated antibody. Membranes were then rinsed and tagged with Goat Anti-Mouse Antibody IRDye 800 CW (LI-COR P/N: 926–32210) and Goat Anti-Rabbit IRDye 680 LT Antibody 690 (LI-COR P/N: 926–68021). All secondary antibodies were diluted 1:10,000. The membranes were visualized using a LI-COR Odyssey Machine. Western data processing is done with LI-COR Image Studio software and GraphPad Prism.

Antibodies used were ROS1 (69D6) Mouse mAb (3266S), p-ROS1 (Y2274) Rabbit Ab (3078S), ERK1/2 p44/p42 MAPK (L34F12) Mouse mAb (4696S), p-ERK1/2 T202/Y204 (197G2) Rabbit mAb (4337S), Akt (pan) (40D4) Mouse mAb (2920S), p-Akt S473 (D9E) Rabbit mAb (4060S) from Cell Signaling Technologies, and Mouse mAb GAPDH (65C) from Millipore (CB1001). pROS1 and ROS1 antibodies were diluted to 1:1000 and all other Cell Signaling Technology antibodies were diluted to 1:2000. GAPDH was diluted to 1:105.

Uncropped and unprocessed scans of the manuscript western blots are shown in Supplementary Fig. 1. All blots were derived from the same experiment and were processed in parallel.

### Reporting summary

Further information on research design is available in the Nature Research Reporting Summary linked to this article.

## Supplementary information

Supplementary Figures

REPORTING SUMMARY

## Data Availability

All the data and resources generated for this study are available in the article or from the corresponding author upon request. The data have been deposited with links to BioProject accession number PRJNA909827 in the NCBI BioProject database (https://www.ncbi.nlm.nih.gov/bioproject/). BioSample metadata are available in the NCBI BioSample database (http://www.ncbi.nlm.nih.gov/biosample/) under accession numbers SAMN32100022, SAMN32100023, and SAMN32100024.

## Code availability

Bioinformatics pipeline code is available upon request.
