# Supplementary material for: Evolution of acquired resistance in a ROS1+ KRAS G12C+ NSCLC through the MAPK pathway
Source: NPJ Precis Oncol. 2023 Jan 23;7:9. doi: 10.1038/s41698-023-00349-0 (PMC9871013; doi:10.1038/s41698-023-00349-0)
Supplement: Supplementary file 1 — Supplementary Figures [file 41698_2023_349_MOESM1_ESM.pdf]

Supplementary Figure 1. Uncropped western blots from **a** Figure 2e and **b** Figure 2f

a.

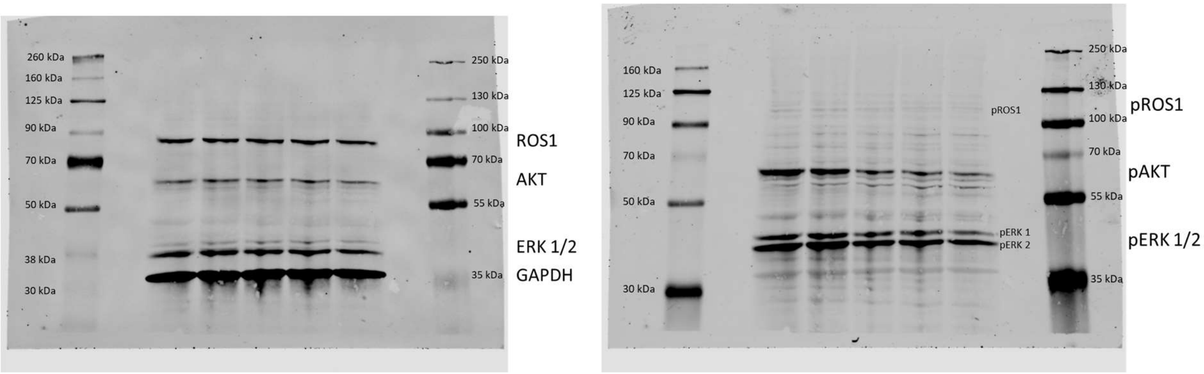

b.

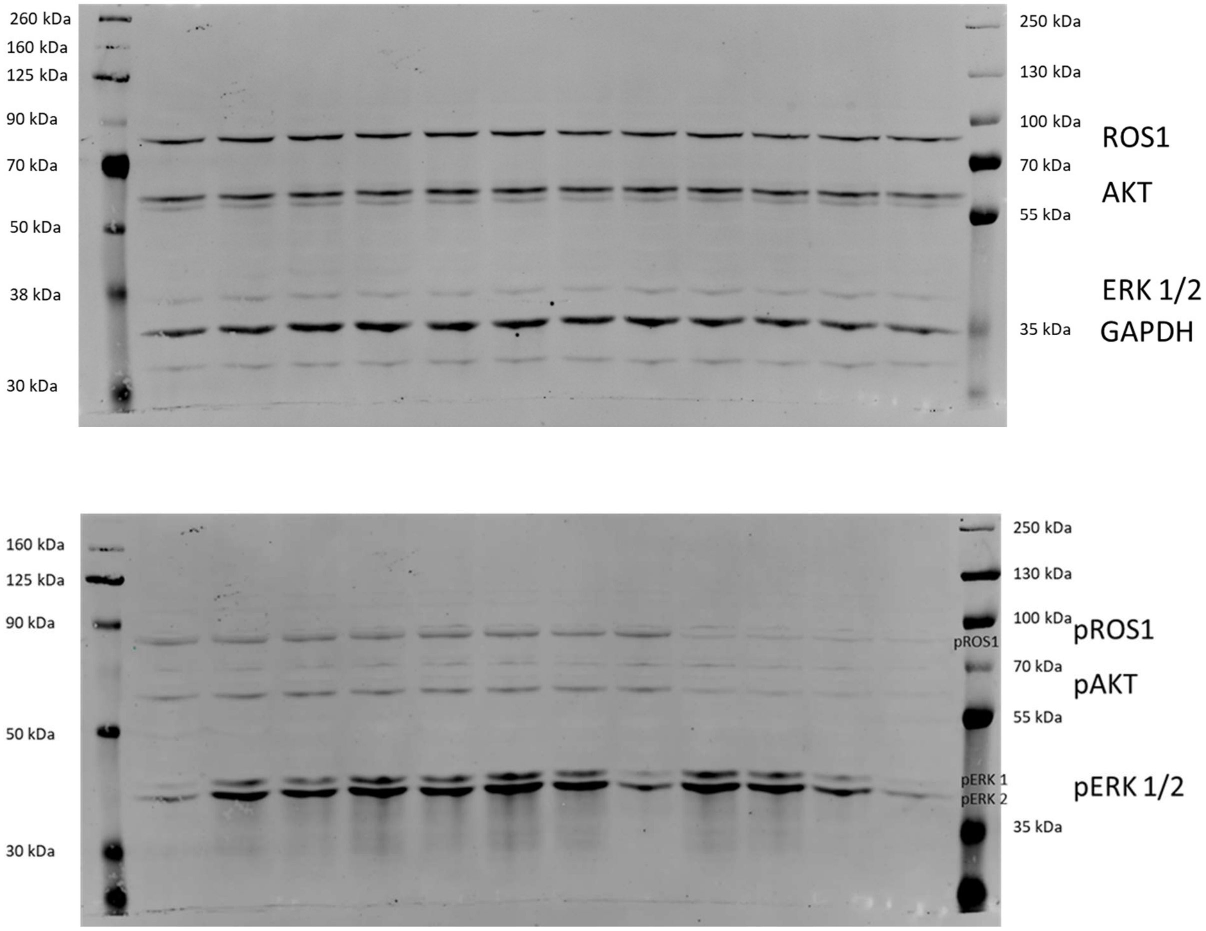

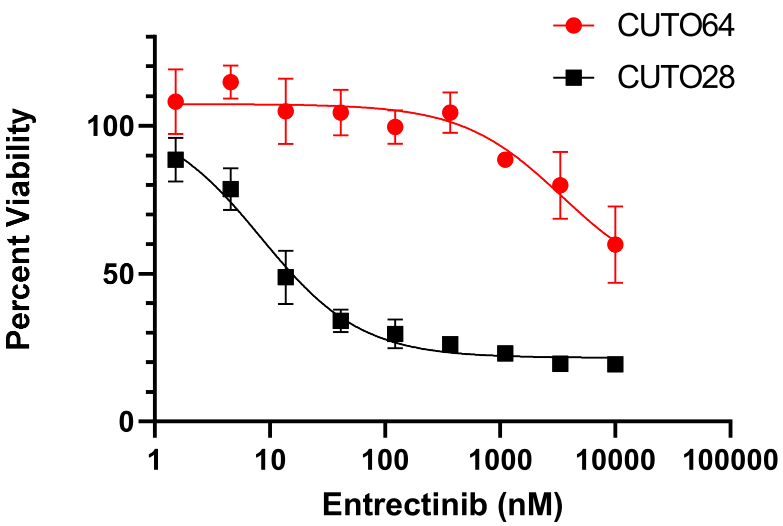

Supplementary Figure 2. TMP3-ROS1 fusion driven NSCLC cell lines can be responsive to TKI. CUTO28 (TMP3-ROS1) is responsive to entrectinib with an IC_{50} of 8.3 nM. CUTO64 (TMP3-ROS1) demonstrates resistance to entrectinib with an IC_{50} > 2000 nM. Showing the mean ± SD.
